# Eradication: Ridding the World of Diseases Forever?

**DOI:** 10.3201/eid1807.120474

**Published:** 2012-07

**Authors:** Jeffrey P. Koplan

**Affiliations:** Emory University, Atlanta, Georgia, USA

**Keywords:** eradication, Nancy Leys Stepan, eradication: ridding the world of diseases forever? Fred Soper, malaria, yellow fever, smallpox, book review

Public health, like any dynamic field filled with social reformers, scientists, and passionate believers, generates conflicting views, approaches, and goals. Thus, on domestic and global fronts, public health advocates compete for priority and resources for vertical (single-disease) versus horizontal (infrastructure or systems) programs; infectious diseases versus noncommunicable diseases; targeting diseases to improve health versus emphasizing the role of economic development or social determinants; and primary health care versus eradicating diseases.

Eradication: Ridding the World of Diseases Forever? by Nancy Leys Stepan provides a rich context for the role of eradication historically and conceptually in public health and, along the way, touches on many of the fault lines that stress and enrich public health. The depth and breadth of the author’s approach also enrich her book and broaden its appeal to readers whose interests go beyond the topic of disease eradication and include public health history, governance, leadership, philosophy, and dependence on multiple disciplines.

The book’s introduction and first chapter alone would provide a fine primer to begin the exploration of “what makes a population get healthier?” After this concise and clear context of eradication and its pursuit (eradicationism), the text then focuses specifically on eradication efforts and some key disease eradicators. Particular emphasis is given to a major 20th century public health leader and proponent of disease eradication, Fred Lowe Soper, and his role with the Rockefeller Foundation, his successful efforts in Brazil and other countries, and his global influence as director of the Pan American Health Organization. He targeted yellow fever and malaria, primarily through vector control (mosquito eradication), and became a champion for use of DDT. Stepan uses the colorful and compelling personality and strengths of Soper, the political complexities of international work, and the unforeseen conflict of insecticidal vector control with the advent of environmentalism to illustrate the considerable hurdles involved in any program of disease eradication, no matter how initially successful and promising. She continues with detailed examples of the successful program of smallpox eradication.

After a description of the guinea worm eradication program, which has made extraordinary progress, the book seems to end a bit abruptly. Only a handful of pages are devoted to the world’s major current disease eradication program, polio, and there is little mention of measles. The book relies for information and opinion on distinguished leaders in eradication efforts, but almost all of them are American or live in the United States. Are European views different? What about having more insights from public health figures in the involved nations in Africa, southern Asia, and South America? The result feels somewhat parochial and incomplete.

Nevertheless, this book provides an interesting and useful perspective on a major public health movement and is suitable for students beginning their public health studies as well as for their professors of epidemiology and public policy. Veterans of eradication efforts will enjoy reading it. Those currently involved in eradication campaigns and those considering joining them would be wise to read this book and absorb its lessons.

